# VSTM1/SIRL‐1: An Inhibitory Pattern Recognition Receptor Regulating Myeloid Cells

**DOI:** 10.1002/eji.202451465

**Published:** 2025-02-24

**Authors:** Maaike Koops, Linde Meyaard

**Affiliations:** ^1^ Center of Translational Immunology University Medical Center Utrecht Utrecht University Utrecht The Netherlands; ^2^ Oncode Institute Utrecht The Netherlands

**Keywords:** inhibitory receptors, pattern recognition receptors, SIRL‐1, VSTM1

## Abstract

Innate immune cells express a plethora of inhibitory receptors, many of which recognize molecular patterns. An appropriate balance between signaling via activating and inhibitory pattern recognition receptors is important for a proper immune response while preventing immunopathology. V‐set and transmembrane domain containing 1 (VSTM1), also known as signal inhibitory receptor on leukocytes‐1 (SIRL‐1), is an inhibitory receptor expressed on myeloid cells. VSTM1 can modulate the function of myeloid cells, by inhibiting reactive oxygen species and neutrophil extracellular trap formation. VSTM1 recognizes shared molecular patterns both from endogenous and microbial origin, defining it as an inhibitory pattern recognition receptor. VSTM1 is involved in various pathological conditions, including autoimmune disorders and cancer, and its restricted expression on myeloid cells highlights its potential as a specific therapeutic target. This review summarizes the characteristics and function of VSTM1 in health and disease.

## Graphical abstract

VSTM1 is an inhibitory receptor expressed on myeloid cells recognizing endogenous and microbial ligands, classifying it as a pattern recognition receptor. During homeostasis, VSTM1 inhibits Fc‐mediated reactive oxygen species (ROS) and neutrophil extracellular trap formation (NET) in granulocytes and monocytes. After activation of the Fc receptor by, for example, an infection, VSTM1 is downregulated and the activating effect outweighs the inhibitory effect.

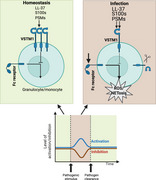

## 1 Introduction

Regulation of adaptive immune cells by inhibitory receptors, such as programmed cell death protein 1 (PD‐1) and cytotoxic T‐lymphocyte associated protein 4 (CTLA4) on T cells, is well studied and successfully therapeutically exploited in cancer and autoimmunity [1, 2]. Likewise, innate immune cells express a plethora of inhibitory receptors [3]. To sense pathogenic signals, innate cells express pattern recognition receptors (PRRs) [4, 5]. PRRs recognize both pathogen‐associated molecular patterns (PAMPs), which are highly conserved components of microbes, and endogenous molecules released by damaged cells, called damage‐associated molecular patterns (DAMPs). The presence of DAMPs and PAMPs should not always result in an immune response. For example, PAMPs are also expressed by commensal bacteria in the skin and gut, which are not necessarily harmful and could even provide benefits to the host. Thus, additional mechanisms need to be in place to regulate immune cells, one of which is inhibitory receptors, which can deactivate signals from activating receptors, resulting in dampening of immune cell activation. Various inhibitory receptors recognize PAMPs and DAMPs and can therefore be classified as inhibitory PRRs [6]. Inhibitory PRRs thus function as the inhibitory counterparts of activating PRRs and provide context to activating signals given by PAMPs or DAMPs. The specific expression pattern of an inhibitory receptor and its ligands will determine in which situation the inhibitory receptor functions. A relatively unknown inhibitory receptor for both microbial and endogenous molecular patterns is VSTM1, also known as SIRL‐1 [7]. In this review, we discuss the characteristics and function of the myeloid‐specific inhibitory receptor VSTM1 in health and disease.

## 2 *VSTM1* Gene and Structure

**FIGURE 1:**
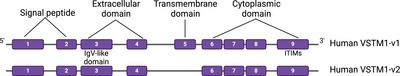
The functional splice variants of the VSTM1 gene. In humans, two different functional splice variants are known, of which VSTM1‐v1 is the predominant variant. Human VSTM1‐v1 exists in 9 exons, while human VSTM1‐v2 lacks the transmembrane domain encoding exon 5. Exon 3 encodes the IgV‐like domain and exon 9 encodes two ITIMs. The figure is created with BioRender.

VSTM1 is encoded by the *VSTM1* gene and was identified in 2010 [7]. *VSTM1* is located close to the Leukocyte Receptor Complex (LRC) region on chromosome 19q13.4, which contains many genes of the immunoglobulin (Ig) superfamily. *VSTM1* exhibits seven different splice variants [8]. Two of these encode the VSTM1‐v1 and ‐v2 proteins that seem to be functionally predominant forms [9]. VSTM1‐v1 is the canonical isoform and is also known as the VSTM1/SIRL‐1 receptor. VSTM1‐v2 differs from VSTM1‐v1 by lacking the transmembrane domain (Figure 1) and is a soluble variant of VSTM1. VSTM1‐v1 is a type I transmembrane glycoprotein that contains one extracellular IgV domain and therefore belongs to the Ig superfamily. Similar to many other inhibitory receptors, VSTM1 contains two immunoreceptor tyrosine‐based inhibitory motifs (ITIMs) in its intracellular tail. The consensus sequence of an ITIM is V/L/I/SxYxxV/L/I, where x denotes any amino acid. The second ITIM of VSTM1 has an atypical sequence (HxYxxL) but contributes to the inhibitory function of SIRL‐1 [7].

## 3 *VSTM1* Gene is not Highly Conserved

**FIGURE 2:**
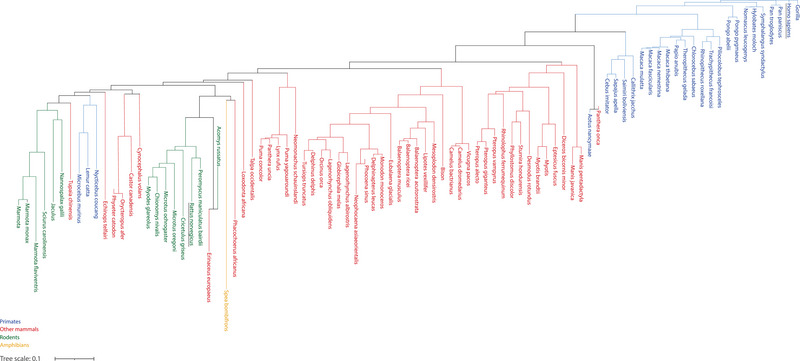
Phylogenetic tree of VSTM1. The figure is based on Ensembl ortholog set 284415.

The coding region of the *VSTM1* gene is conserved in around 100 different species (Figure 2) [10]. In rats, the *VSTM1* gene has recently been identified and seems to show similar inhibitory functions as in humans, despite the fact that rat VSTM1 does not have an ITIM motif and instead has a repeat glutamic acid‐rich protein sequence [11]. The homology between rat and human *VSTM1* is merely 48% while, for instance, chimpanzee and human *VSTM1* show 98% homology [10]. In mice, no functional VSTM1 protein has been identified. The mouse *VSTM1* gene is homologous to rat *VSTM1* but has a premature stop codon in exon 2 and multiple in‐frame stop codons in other exons and is presumably degenerated to a pseudogene [11]. Therefore, studying VSTM1 in mouse models would require a transgenic or humanized system, which currently have not been reported. Inhibitory receptors that lack sequence homologues in mice, may have functionally analogous proteins. For example, in mice, the c‐type lectin Ly49 family of inhibitory receptors perform a similar role to the structurally completely different human Killer Ig‐Like Receptors (KIRs) [12]. It remains to be determined whether a functionally analogous protein for VSTM1 is encoded in animal genomes without functional VSTM1.

## 4 VSTM1 Recognizes Multiple Endogenous and Microbial Ligands

The first identified ligands for VSTM1 are S100 proteins [13]. S100 proteins are small proteins characterized by two calcium‐binding sites [14]. Some S100s are DAMPs that interact with activating receptors and thereby promote inflammation. In contrast, binding of S100 proteins to VSTM1 results in inhibitory signals [13]. The antimicrobial peptide LL‐37, which is secreted by epithelial cells, is another DAMP recognized by VSTM1 [15]. DAMPs may thus provide both activating and inhibitory signals to the immune cell, possibly as a negative feedback mechanism to prevent overactivation or to prevent activation by harmless stimuli, such as commensal bacteria. Recently, galectin‐1 was additionally identified as a ligand for VSTM1 [16]. Galectins are proteins that bind to β‐galactoside sugars and play a role in various biological processes, such as cell survival and migration [16, 17]. Besides endogenous ligands, VSTM1 also recognizes staphylococcal phenol‐soluble modulins (PSMs), which are peptides produced by staphylococci and can thus be regarded as PAMPs. PSMs function as cytotoxins and pro‐inflammatory agents and are structurally related to the LL‐37 peptide. Activation of inhibitory signaling by PAMPs is not unique to VSTM1 [18, 19]. For instance, the inhibitory Siglec receptors recognize sialic acids, which are also present on the surface of (opportunistic) group B streptococcus [20]. There are several views as to why a microbial ligand would interact with an inhibitory receptor. On the one hand, it could be a pathogenic strategy for immune evasion and thus detrimental to the host [18, 19]. However, the interaction between an inhibitory receptor and a microbial ligand might also prevent immune activation in response to a harmless commensal microbe or when the damage caused by the immune response outweighs the damage caused by the microbe [18, 19]. In this way, recognition of microbial patterns by inhibitory receptors could also be beneficial for a commensal lifestyle.

## 5 What is Needed for VSTM1 Signaling?

**FIGURE 3:**
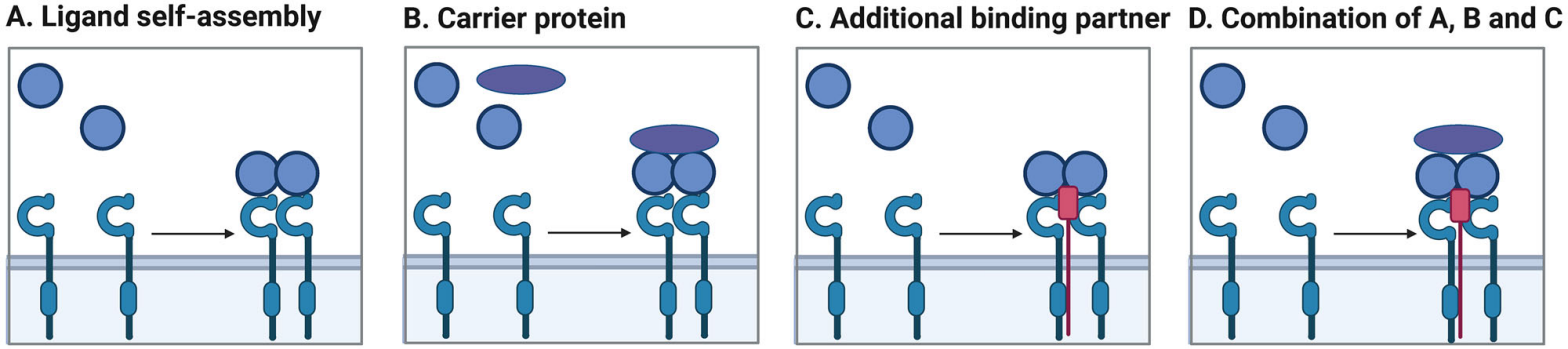
Hypothetical mechanisms for VSTM1 clustering. Clustering might be induced by ligand self‐assembly into an oligomer (A), by binding several ligands to a scaffold (B), by a co‐receptor (C), or by a combination of these mechanisms (D). The figure is created with BioRender.

Activation of inhibitory receptors typically leads to phosphorylation of the tyrosine within the ITIM by Src family kinases [21, 22]. This phosphorylation allows docking and activation of inhibitory effectors that contain a Src homology 2 (SH2) domain, such as SH2 domain‐containing phosphatases 1 and 2 (SHP‐1, SHP‐2) or C‐terminal Src kinase (Csk). These effectors deactivate the signaling components of activating receptors. In the case of VSTM1, both ITIMs are needed to recruit SHP‐1 and SHP‐2 to relay its inhibitory signals [7]. Next to SHP‐1 and SHP‐2, possibly other molecules are recruited by VSTM1, since mutation of tyrosine‐to‐phenylalanine in the ITIMs does prevent SHP‐1 or SHP‐2 recruitment but still results in partial inhibition. The mechanism through which VSTM1 ligands activate VSTM1 is unclear. In contrast to galectin‐1, no direct binding to VSTM1 has been detected for S100s, LL37, or PSM yet, neither by cell‐based assays nor assays with purified recombinant proteins [13, 15]. There might therefore be an additional binding partner required. Activation of inhibitory receptors is generally thought to require clustering of the receptor [23, 24], which allows phosphorylation by Src‐family kinases [25]. The induction of clustering could be induced by the self‐assembly of ligands into oligo‐ or polymers, by sequestering of ligands by a carrier protein, or by a co‐receptor needed for stabilization (Figure 3) [26, 27]. VSTM1 ligands PSMs, LL37, and s100 proteins are known to self‐assemble or form fibrils and might in this way provide VSTM1 clustering [28, 29, 30]. Furthermore, PSMs and LL‐37 can interact with extracellular DNA or neutrophil extracellular traps (NETs) and thereby form scaffolds [31, 32, 33]. Whether galectin‐1 could be involved in such a complex is currently unclear.

## 6 VSTM1 is a Regulator of Granulocytes and Monocytes

VSTM1 is exclusively expressed in myeloid cells, including neutrophils, eosinophils, and monocytes [34]. All granulocytes express VSTM1 in blood and tissue (blood, colon, and lung), whereas VSTM1 expression in monocytes is tissue‐specific. VSTM1 is not expressed in monocytes in the skin and colon, while lung monocytes express high levels of VSTM1 [34]. Among the monocyte subsets in both blood and lung, VSTM1 expression is most prominent in classical monocytes and less expressed in nonclassical monocytes. VSTM1 inhibits both Fc receptor (FcR)‐induced reactive oxygen species (ROS) in neutrophils and monocytes and NET formation in neutrophils [13, 16, 35, 36]. Inhibition of NET formation by VSTM1 results in reduced extracellular killing of bacteria, but VSTM1 does not affect phagocytosis and intracellular bacterial killing [35, 37].

## 7 VSTM1 Expression Decreases After Cell Activation

**FIGURE 4:**
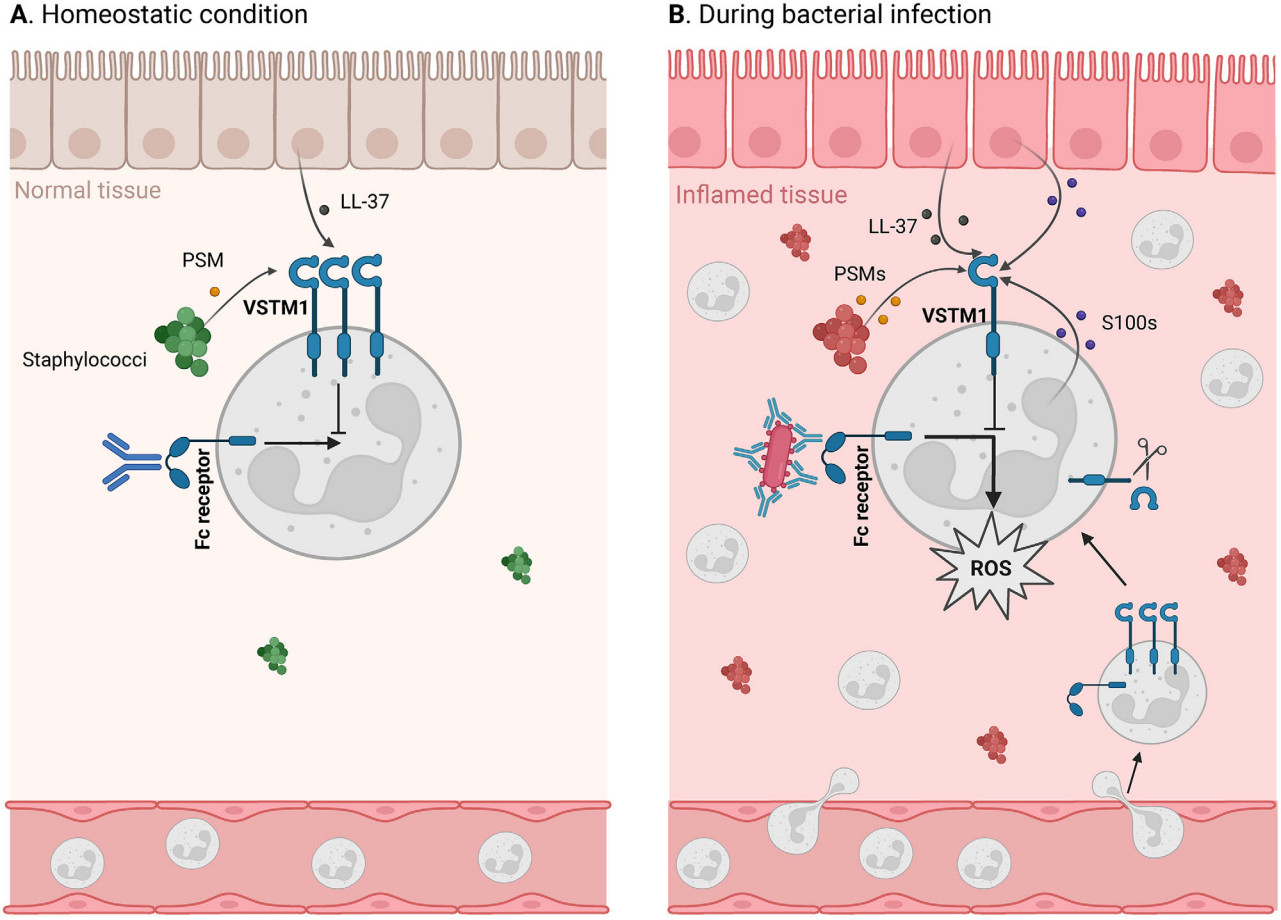
Possible model for VSTM1 function. (A) In homeostasis, VSTM1 on phagocytes prevents inappropriate activation, for example, induced by nonspecific antibody stimulation of the Fc receptor. VSTM1 stimulation might be induced by the presence of commensal bacteria secreting phenol‐soluble modulin (PSM) or low levels of endogenous ligands such as galectin‐1, S100, or LL37. (B) During a bacterial infection, neutrophils are recruited to the site of infection and VSTM1 expression decreases, enhancing reactive oxygen species (ROS) production to facilitate optimal microbial killing. Upon tissue damage, endogenous VSTM1 ligands (PSMs, LL‐37, s100s) are secreted in high amounts and even low VSTM1 expression might, therefore, still be able to inhibit Fc receptor‐induced ROS production to prevent further tissue damage. The figure is created with BioRender.

The regulation of expression of inhibitory receptors is an essential mechanism to maintain immune homeostasis and prevent autoimmunity. Inhibitory receptors can be divided into categories based on their expression [38]. Threshold receptors are continuously expressed and set a threshold to prevent immune activation in response to harmless stimuli. Negative feedback receptors are absent on resting cells but are upregulated after activation to provide negative feedback to terminate immune responses. Lastly, disinhibition receptors are consistently expressed but are downregulated after activation of the cell [38]. The expression of VSTM1 depends on the activation status of the cell. Microbial and inflammatory stimuli cause rapid downregulation of VSTM1 on granulocytes and monocytes in vitro [7]. In respiratory syncytial virus (RSV) infection, the expression of VSTM1 in sputum neutrophils is decreased compared with its expression in peripheral blood neutrophils [35]. Thus, VSTM1 probably acts as a disinhibition receptor on granulocytes. When no or low immune cell activation is warranted, VSTM1 can prevent excessive responses and limit immunopathology, while VSTM1 is downregulated in case a stronger cellular response is needed. However, VSTM1 expression is not completely lost upon cellular activation [35]. The remaining VSTM1 might still result in inhibition of granulocytes, particularly when inflammation has already caused considerable tissue damage leading to high concentrations of ligands (Figure 4).

The downregulation of VSTM1 is mediated by ectodomain shedding by proteolytic cleavage with proteinase 3 which occurs rapidly after cell activation [39]. In patients with RSV or COVID‐19 infection, soluble VSTM1 is increased in sputum compared with plasma, suggesting that VSTM1 is shed from the cell surface during infection [39]. In contrast, *Staphylococcus aureus* can prevent VSTM1 shedding by the extracellular adherence protein (Eap) which inhibits proteinase 3, potentially representing an immune‐escape mechanism [39].

Thus, VSTM1 might set a threshold to control activating signals, subsequently be downregulated to facilitate consequent immune activation, but possibly still control myeloid cells in the presence of high amounts of ligands.

Next to ectodomain shedding, there might be a second source of soluble VSTM1. As mentioned before, the splice variant VSTM1‐v2 lacks the transmembrane domain and could function as a secreted glycoprotein. One study showed that VSTM1‐v2 can promote the differentiation and activation of Th17 cells, in contrast to the function of VSTM1‐v1 [9]. However, we could not reproduce these experiments to support a role for VSTM1‐v2 in Th17 cell differentiation [40]. In addition, the in vivo presence of endogenous soluble VSTM1 protein encoded by VSTM1‐v2 has not been demonstrated yet [39]. The presence of soluble VSTM1 in circulation in vivo may thus solely arise from the shedding of the VSTM1 ectodomain.

## 8 VSTM1 Might Play a Role in Autoimmune Diseases

Autoimmune diseases reflect an aberrant immune response, resulting in inflammation of various organ systems [41]. The role of inhibitory receptors in autoimmune diseases is unclear, but there are multiple lines of evidence for a link between inhibitory receptors and the predisposition to autoimmune diseases [42]. For example, inhibitory receptor knockout mice are more prone to develop autoimmune pathology, there is a genetic association of multiple inhibitory receptors and autoimmunity, and blockade of inhibitory receptors in the context of cancer results in autoimmune‐like symptoms [42, 43]. The role of VSTM1 in autoimmune diseases is primarily investigated through its role in regulating ROS production and NET formation in autoimmune diseases, such as systemic lupus erythematosus (SLE) and rheumatoid arthritis (RA) [44, 45, 46, 47]. SLE is a chronic heterogenous multisystemic autoimmune disease, characterized by the accumulation of antinuclear antibodies. RA is a chronic autoimmune disease that primarily involves synovial joints, characterized by rheumatoid factor and anti‐citrullinated protein antibodies. Similar to healthy phagocytes, activation of VSTM1 inhibits FcR‐induced ROS production and NET formation in phagocytes of SLE and RA patients in vitro [16, 35, 36, 37, 48].

As described earlier, VSTM1 can be downregulated after inflammatory stimuli. In RA, VSTM1 expression is decreased on neutrophils in blood and synovial tissues compared with healthy controls [49]. In addition, soluble VSTM1 is increased in serum and synovial fluid from RA patients compared with healthy controls or osteoarthritis patients, respectively [50]. The concentration of soluble VSTM1 is higher in patients with active disease compared with patients in remission. Also, VSTM1‐v2 mRNA expression in RA patients is increased compared with healthy controls [51]. In SLE, no difference in expression of VSTM1 on neutrophils from SLE patients compared with healthy controls was observed by our group [37], but another study found decreased VSTM1 expression on SLE neutrophils [16]. Reduced expression of VSTM1 and increased levels of its soluble form in autoimmune disease are not unique to VSTM1; other inhibitory receptors also exhibit similar changes, such as decreased expression of programmed cell death protein 1 (PD‐1) and increased soluble forms of leukocyte‐associated immunoglobulin‐like receptor 1 (LAIR‐1) in SLE or RA [42, 52].

**FIGURE 5:**
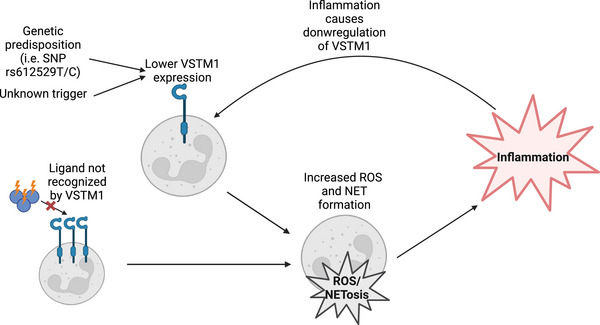
Possible roles of VSTM1 in inflammation/autoimmunity. Genetic predisposition, i.e., SNP rs612529T/C, or an unknown trigger can result in decreased expression of VSTM1, resulting in increased reactive oxygen species (ROS) and neutrophil extracellular trap (NET) production. Alternatively, VSTM1 ligands could be modified during inflammation and not recognized by VSTM1, also resulting in increased ROS and NET formation. In addition, inflammation itself also results in downregulation of VSTM1. The figure is created with BioRender.

Aberrant VSTM1 function could contribute to disease in several ways (Figure 5). Downregulation of VSTM1 might result in increased ROS production and NET formation and therefore contribute to the pathogenesis of these diseases. Another possibility is that inflammation‐related modifications to VSTM1‐ligands could prevent proper inhibition by VSTM1. One study found that in SLE the VSTM1 ligand galectin‐1 is oxidized and can therefore not be recognized by VSTM1 [16]. This might contribute to dysregulated neutrophil function with increased ROS production and NET formation in SLE. Lastly, a single nucleotide polymorphism (SNP), rs612529T/C, has been identified in the *VSTM1* gene [34, 53]. This SNP is located in the promoter region and mediates the epigenetic silencing of the VSTM1 gene. This process is presumably mediated by allele‐specific binding of transcription factor PU.1 to the promoter region of VSTM1. Individuals homozygous for this SNP have an almost total absence of VSTM1 expression specifically on their monocytes, which precludes VSTM1‐mediated inhibition of FcR‐induced ROS production. This SNP is associated with atopic dermatitis (AD), a chronic inflammatory skin disease. Decreased VSTM1 expression in individuals with this SNP might lead to increased ROS production which could contribute to the pathogenesis of AD. Although healthy skin monocytes have low VSTM1 expression [34], monocytes newly recruited to the skin probably still express VSTM1, and the absence of VSTM1 expression in this time frame might predispose to AD. Of interest, AD patients are often colonized with *S. aureus*, which expresses the VSTM1 binding PSMs.

## 9 VSTM1 and Atherosclerosis

Inflammation plays an important role in atherosclerosis, particularly through the involvement of monocytes and macrophages [54]. Monocytes accumulate into vascular endothelial cells and turn into macrophages through the phagocytosis of oxidized low‐density lipoprotein (ox‐LDL). The role of VSTM1 in this disease has been examined by two studies. In a cohort with patients with coronary heart disease, low VSTM1 expression on monocytes is a prognostic factor for major cardiac events [55]. In vitro depletion of VSTM1 in monocytes stimulated by ox‐LDL results in increased ROS production, increased invasiveness and chemotaxis of monocytes, and reduced cell apoptosis [54]. Reduced VSTM1 might therefore result in increased monocyte‐mediated inflammation in atherosclerosis.

## 10 VSTM1 and Cancer

Tumor cells often have an increased expression of inhibitory ligands to evade the immune system and are therefore used as a target for immunotherapy. A few studies investigated VSTM1 in cancer. One study examined the gene expression of VSTM1 in hematopoietic tumor cell lines [56]. VSTM1 is silenced in most of the examined hematopoietic tumor cell lines as a result of promoter methylation. This is in contrast to other inhibitory receptors, such as PD‐1 and LAIR‐1, which are upregulated in hematopoietic malignancies [57, 58]. Downregulation of VSTM1 thus seems favorable for a hematopoietic tumor cell, the mechanism of which is still unclear. Stimulation of VSTM1 results in the inhibition of leukemic cell growth [56, 59], and VSTM1 overexpression through an oncolytic adenovirus in leukemic cells in vitro has antiproliferative and proapoptotic effects on leukemic cells, which was less pronounced in healthy cells [60]. Although the reason why this effect is less pronounced in healthy cells compared with leukemic cells is unclear, this is suggestive of VSTM1 as a specific therapeutic target for leukemia. One study investigated VSTM1 RNA expression in solid tumors [11]. In advanced‐stage lung cancer, VSTM1 expression on tumor‐associated myeloid cells was increased compared with early‐stage lung cancer, suggesting VSTM1 expression on myeloid cells increases during tumor development. This is similar to most other inhibitory receptors whose expression also increases during cancer progression, resulting in immune cell exhaustion and a reduced ability to effectively eliminate tumor cells [1].

## 11 Conclusion

In conclusion, VSTM1 is an inhibitory receptor expressed on myeloid cells which is downregulated after infectious and inflammatory stimuli, categorizing it as a disinhibition receptor. Multiple functional ligands of VSTM1 have been identified of both endogenous and microbial origin, defining VSTM1 as an inhibitory PRR. However, the mechanism by which these ligands bind and activate VSTM1 is unclear. Due to the lack of a mouse model and the availability of only one commercially available antibody, studying VSTM1 may be challenging, but the number of labs studying this receptor is increasing. In disease, VSTM1 is differentially expressed and might play a role in various autoimmune diseases and cancer. Whether aberrant VSTM1 function serves as a predisposing factor for autoimmune diseases or whether it is a result of sustained inflammation within autoimmune conditions remains unclear. Nevertheless, VSTM1 is an interesting target for these diseases. Agonists for other inhibitory receptors are currently undergoing the first clinical trials to assess their impact on autoimmune diseases [61]. Given its restricted expression, further investigation into the potential of VSTM1 agonists is warranted. In cancer therapy, VSTM1 is currently scarcely explored. Since many patients are either resistant to or relapse after current checkpoint inhibitors, VSTM1 therapy could be considered as a candidate for combination therapy. As current therapies focus on targeting inhibitory receptors on T cells, addressing VSTM1 expressing myeloid cells may unleash therapeutic potential through another mechanism.

## Conflicts of Interest

L. M.’s research laboratory at UMC Utrecht has received research funding from NextCure, NGM Biopharmaceuticals, Boehringer Ingelheim, and argenx and has received consultancy fees from Eli Lilly, Third Rock Ventures, and Abbvie. L. M. has not received personal fees or other personal benefits. The remaining author declares no conflicts of interest.

### Peer Review

The peer review history for this article is available at https://publons.com/publon/10.1002/eji.202451465.

## Data Availability

Data sharing is not applicable to this article as no datasets were generated or analyzed during the current study.
